# Clinical Data Flow in Botswana Clinics: Protocol for a Mixed-Methods Assessment

**DOI:** 10.2196/52411

**Published:** 2024-10-09

**Authors:** Grey Faulkenberry, Audrey Masizana, Badisa Mosesane, Kagiso Ndlovu

**Affiliations:** 1 Department of Biomedical and Health Informatics Children's Hospital of Philadelphia Philadelphia, PA United States; 2 eHealth Research Unit Department of Computer Science University of Botswana Gaborone Botswana; 3 Global Health Informatics Children's Hospital of Philadelphia Gaborone Botswana; 4 Informatics Division Sir Ketumile Masire Teaching Hospital Gaborone Botswana

**Keywords:** global health, health information systems, electronic health care records, EHRs, interoperability, data flow, access to information, health information interoperability, pediatric, pediatrics, infant, infants, clinical data, mixed method, Botswana, health care information, child health, tuberculosis, HIV, eLearning, survey, health informatics, communication

## Abstract

**Background:**

Botswana has made significant investments in its health care information infrastructure, including vertical programs for child health and nutrition, HIV care, and tuberculosis. However, effectively integrating the more than 18 systems in place for data collection and reporting has proved to be challenging. The Botswana Health Data Collaborative Roadmap Strategy (2020-24) states that “there exists parallel reporting systems and data is not integrated into the mainstream reports at the national level,” seconded by the Botswana National eLearning strategy (2020), which states that “there is inadequate information flow at all levels, proliferation of systems, reporting tools are not synthesized; hence too many systems are not communicating.”

**Objective:**

The objectives of this study are to (1) create a visual representation of how data are processed and the inputs and outputs through each health care system level; (2) understand how frontline workers perceive health care data sharing across existing platforms and the impact of data on health care service delivery.

**Methods:**

The setting included a varied range of 30 health care facilities across Botswana, aiming to capture insights from multiple perspectives into data flow and system integration challenges. The study design combined qualitative and quantitative methodologies, informed by the rapid assessment process and the technology assessment model for resource limited settings. The study used a participatory research approach to ensure comprehensive stakeholder engagement from its inception. Survey instruments were designed to capture the intricacies of data processing, sharing, and integration among health care workers. A purposive sampling strategy was used to ensure a wide representation of participants across different health care roles and settings. Data collection used both digital surveys and in-depth interviews. Preliminary themes for analysis include perceptions of the value of health care data and experiences in data collection and sharing. Ethical approvals were comprehensively obtained, reflecting the commitment to uphold research integrity and participant welfare throughout the study.

**Results:**

The study recruited almost 44 health care facilities, spanning a variety of health care facilities. Of the 44 recruited facilities, 27 responded to the surveys and participated in the interviews. A total of 75% (112/150) of health care professionals participating came from clinics, 20% (30/150) from hospitals, and 5% (8/150) from health posts and mobile clinics. As of October 10, 2023, the study had collected over 200 quantitative surveys and conducted 90 semistructured interviews.

**Conclusions:**

This study has so far shown enthusiastic engagement from the health care community, underscoring the relevance and necessity of this study’s objectives. We believe the methodology, centered around extensive community engagement, is pivotal in capturing a nuanced understanding of the health care data ecosystem. The focus will now shift to the analysis phase of the study, with the aim of developing comprehensive recommendations for improving data flow within Botswana's health care system.

**International Registered Report Identifier (IRRID):**

DERR1-10.2196/52411

## Introduction

### Background

Over 2500 years ago, Hippocrates argued it was important to keep a record of a patient’s conditions and symptoms [1,2]. While we have developed new ways of record keeping, accurate and timely record keeping remains as important, if not more so, given the interconnectedness of communities. The World Health Organization has further emphasized the importance of electronic health records (EHRs) toward the achievement of Universal Health Coverage and health-related sustainable development goals [3]. In the United States and other high-income countries, EHRs capture 90% or more of these data. However, in low- and middle-income countries, the percentage is much smaller but steadily increasing. Despite the documented benefits of EHRs, a number of concerns have been raised. The aim of this study is to assess the flow of clinical data through various systems (EHRs and electronic medical records) by understanding and describing the flow of clinical data. In addition, the study explores the perceptions of health care workers regarding health care data collection, sharing, and use across Botswana’s health care facilities.

### Data Generators

Data are often collected using multiple separate systems and reported in aggregate at a system or national level, with varying benefits seen by frontline clinicians [4]. This is more than simply an aggravation. Ensuring that frontline clinicians have proper access to data could improve clinical outcomes on a wide range of issues, from the correct treatment of pediatric malaria [5] to decreasing medication errors in tuberculosis treatment and even reducing patient wait times in clinics [6].

From a systems perspective, lack of access to data, concerns about data validity and accuracy, perceived uselessness of data collection, and an inability to transfer information are continually ranked among the leading barriers to effectively implementing EHRs by clinical staff, administrators, medical directors, and information technology personnel [7,8]. A study conducted in South Africa [9] emphasized that data routinely collected at health care facilities and submitted to district offices are commonly unreliable. The study investigators further asserted that “data validation was limited” and “little analysis of data occurred” at the participating facilities sampled in their study, leading to a gloomy picture overall. However, even if analysis tools were improved, with multiple systems in use, they would only be applicable to a small subset of all the data collected. Another study on the Prevention of Mother to Child Transmission of HIV (PMTCT) in South Africa [10] adds that there are major gaps in both completeness and accuracy in the collection and reporting of data that track service delivery.

Given these concerns, it is crucial to understand the views of those inputting and generating these data. As one study found, “data quality issues are not a result of the type of record, but the attitude of the person inputting the data” [11].

### Interoperability

What is desired is to integrate organizational information systems, devices, and applications to access, exchange, and cooperatively use data across organizations. This would provide a platform where systems with different infrastructures could share data and services and, hence, have the same expectations for the contents, context, and meaning of the data. To advance health information system interactions, there must be interoperability (“the ability of two or more systems or components to exchange information and to use the information that has been exchanged” [12]). Improved interoperability would result in a decrease in missing data as systems fill in each other’s gaps and alleviate some of the clerical burden inherent in using multiple systems. Consequently, there would be improved health data quality [13]. Interoperability is often guided by an interoperability framework, offering an agreed approach for multiple organizations to achieve interoperability toward the joint delivery of services.

Botswana supports noninteroperable diverse health information systems [14] and currently has more than 18 systems in place for data collection and reporting. A 2019 UNICEF report on Botswana highlights these points [15], making note of the different data tracking systems that “are not necessarily coordinated or reliable across health facility levels and systems.” For example, UNICEF highlights Botswana’s strong HIV early infant diagnosis program but notes the long turnaround times for results and lack of HIV-exposed infants’ final infection status. The report further states, “Key PMTCT variables have poor quality data thought to be associated with lack of understanding of the required data and lack of uniformity in recording and reporting” [15]. One of the key recommendations of this report was to “look for opportunities to simplify, harmonize, reduce redundancy and roll out the most reliable systems to all districts and health facilities.”

In response, the Botswana Ministry of Health (MOH) released the Botswana Health Data Collaborative Roadmap Strategy (2020-24) [16], which states that “there exists parallel reporting systems and data is not integrated into the mainstream reports at the national level”, seconded by the Botswana National eLearning strategy (2020) [17], which states that “there is inadequate information flow at all levels, proliferation of systems, reporting tools are not synthesized; hence too many systems are not communicating.”

### Objectives

This study was driven by 2 primary goals to align with the MOH’s objectives of enhancing data collection, data sharing, reporting, and usage: to create a visual representation of data flow within the health care ecosystem of Botswana, and gain insights into how frontline workers perceive the collection, use, and sharing of data with the health care sector.

A data flow diagram would encompass the entire data journey, starting with data generation by the frontline clinician and tracking it through various stages, including data transfers, reporting, administrative processes, laboratory operations, government involvement, and, where applicable, returning it to the frontline workers who generated it. By visualizing this data flow, the study intends to offer insights into how data inform operations at different levels and highlight the exchange of information among people, processes, and system components. Describing and understanding this flow, along with relevant system protocols and boundaries, is crucial for improving decision-making and strategic planning within an organization. Poor data flow could lead to incompatible and inconsistent data systems, resulting in information silos. Conversely, if data linkages are properly established, there is potential to enhance data access and sharing, ultimately leading to valuable insights that exceed the sum of isolated data sources.

Understanding the perceptions of frontline health care workers is essential, particularly in the context of potential system integration and workflow changes. Frontline workers' attitudes toward data collection directly impact how it is used as well as its quality [11]. This goal aims to assess how health care data are currently accessed, transferred, used, and reported by workers, as well as whether they recognize value in these processes.

## Methods

### Setting and Community Engagement

This study commenced to engage health care facilities across the country, with an attempt to engage facilities as widely as possible in each district. The purpose of the sensitizations was to introduce the study, secure buy-in, and increase the participation of health care workers at each site.

The approach to engaging health care facilities involved direct contact with their leadership. The study team reached out through phone calls and formal email invitations, followed by virtual and onsite sensitization meetings and workshops. Over 70 emails were sent out to health care facilities to arrange their involvement in the study, and close to 100 phone calls were made to engage facilities’ leadership to coordinate participants' involvement in the study. These sessions played a crucial role in gaining the facilities' buy-in and motivating their health care workers to participate. To encourage maximum participation and inclusivity, we embraced an open community research approach. Our target population consisted of health care professionals who were directly involved in data collection, data sharing, and data-driven decision-making or used information and communications technology systems for health care tasks. Healthcare workers’ daily experience in data-related tasks made them invaluable contributors to this study. The study team emphasized clinicians’ role as collaborators in the project, underlining the significance of their input in shaping the research instruments. This approach resulted in the refinement of questions in the REDCap (Research Electronic Data Capture; Vanderbilt University) survey instruments and the inclusion of new ones, ultimately leading to more insightful responses.

### Study Design

This study adopts a mixed methods approach to assess the flow of clinical data across Botswana’s health care facilities. The design of the study encompassed both qualitative and quantitative methods to gain a comprehensive understanding of how health care data are collected, shared, and used in the selected facilities. The surveys were informed by the rapid assessment process [18-20] model and the technology acceptance model for resource-limited settings [21,22], ensuring they were both comprehensive and contextually relevant.

### Survey Instruments

Our data collection instruments were developed to align with the study's objectives and to ensure that we could capture a comprehensive view of the health care data flow in Botswana's health care system. During the development, we discovered a notable gap in the existing literature. Previous assessments of e-health readiness and maturity have used various frameworks [23-26] that assess dimensions such as core need readiness, technological readiness, and learning readiness. The MEASURE Evaluation [27] framework emerged as the only assessment we found that documents the flow of clinical data from the patient to the clinician, to the labs or government, and ultimately back to the clinician and patient. Visual representations were essential for enhancing our comprehension, given their widespread use across various health care specialties and industries [28,29].

This resulted in a 2-pronged survey approach. The first was a self-led quantitative digital survey used to broadly understand how clinicians collect data and share it. This method focused on the demographics of the participants, the electronic systems used, data collection mechanisms, frequency of reporting, and usage of the collected health care data.

**Figure 1:**
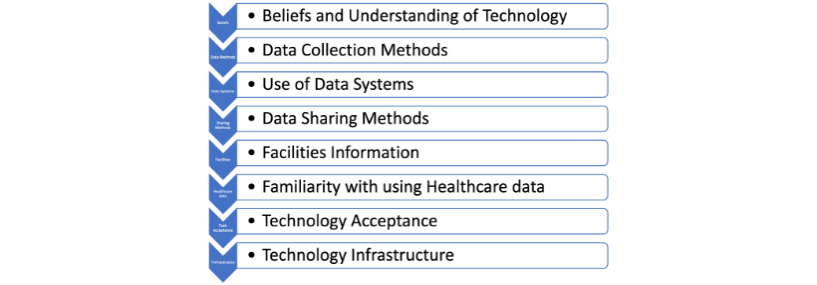
Key features and questions.

The qualitative component of this study involved a semistructured interview method, allowing the participants to offer detailed perceptions on the use of technology and data management practices within health care settings in Botswana. These interviews, conducted by research assistants, were informed by the study’s objectives and relevant literature, focusing on health care workers’ perspectives on collecting and managing health care data and service delivery. Participants, including administrators, nurses, and doctors with various roles in the health care sector, were encouraged to provide in-depth insights into both manual and digital data collection methods, along with their views on data sharing and usage in Botswana. The aim was to elicit comprehensive information and opinions regarding the flow of clinical data, focusing on key features and critical questions outlined in Figure 1.

### Sampling

Purposive sampling was used to select participants who interacted with health care data representing various levels of the health care system, including general nurses, monitoring and evaluation officers, health care administrators, frontline clinicians, and IT professionals. All participants were invited to participate in the 2 surveys of this study.

### Selection Criteria and Representation

We strategically chose health care facilities located at 3 locations in Botswana: Gaborone, Maun, and Selebi Phikwe. Each of these facilities and their surrounding areas represent a distinct region of the country. This choice allowed us to capture diverse perspectives, reflecting the varied health care environments within the country. Ethical approval at the facility level was a paramount prerequisite before the study commenced, with key contacts typically residing within the institutional review board (IRB) chairmanship.

### Data Collection

The REDCap system, together with interviews, was used to collect data from study participants. The surveys were distributed electronically to a representative sample of health care workers across different health care facilities in Botswana. A comprehensive list was compiled for all of the large public health care facilities in the country, leading to invitations being extended to over 30 health care facilities, encompassing both comprehensive public hospitals and clinics. Out of these, 23 sites expressed interest in the study and actively participated in it, with full engagement in sensitization and data collection workshops. About 15 sensitization sessions were held to inform and engage health care facility leadership and health workers about the study’s objectives, methods, and potential impacts and obtain feedback. Of the 15, 4 were held in-person, while 11 were held virtually via Microsoft Teams led by the study team research assistants at the University of Botswana.

A total of 157 responses were collected from the first survey, while the second survey had 227 responses.

### Data Analysis

Data from the quantitative survey will be analyzed using descriptive statistical methods to examine trends, correlations, and associations related to health care data sharing and usage at hospitals and clinics.

We plan to use thematic analysis to analyze the qualitative results of the study, mainly to identify recurring themes, patterns, and insights from the qualitative data provided by the study participants. The analysis will begin by focusing on themes such as perceptions of the value of health care data, experiences of data collection and sharing, and the adoption and familiarity of different technological solutions.

Findings from both qualitative and quantitative data collection will be integrated to provide a comprehensive understanding of the flow of clinical data across the sampled facilities. Data integration will involve comparing and contrasting qualitative themes with quantitative survey results to validate and complement the findings.

For analysis purposes, tools including Jupyter notebooks and NVivo software will be used to pre-process the data and develop code for analyzing the data coming from the participating health care workers.

### Ethical Considerations

Before starting this study, we ensured we had all appropriate permissions from the IRBs at the University of Botswana (protocol or reference number UBR/RES/IRB/BIO/258), the Human Research Development Committee in Botswana, the University of Pennsylvania (protocol number 849993), and the Children’s Hospital of Philadelphia (protocol number 22-019994), in addition to letters of support from the MOH and the local institutions with which we were working. Additionally, ethical approval was obtained at the health facility level through IRB approval and review. Prior to participating, all responders agreed and signed an informed consent. All identifiable data were stored securely in an encrypted cloud server, requiring password access, with regular security auditing.

### Adapting to COVID-19 Restrictions

Acknowledging the constraints imposed by the COVID-19 pandemic, this study was adapted to ensure safety without compromising engagement. Most sensitization sessions and all interviews were conducted remotely, using video conferencing tools to maintain interactive and personal communication channels.

## Results

This study recruited close to 44 health care facilities, including district hospitals, public referral hospitals, primary health care clinics, mobile health clinics, and health posts. Out of the 44 facilities recruited, 27 responded to our surveys and interviews. About 75% of the health care professionals who participated in the study came from clinics, 20% from hospitals, and 5% from health posts and mobile clinics. Our results indicate that 2 essential factors are crucial to participation in our assessment. First, sensitization meetings are followed by stakeholder engagement workshops with participants. Second, establishing an open and collaborative environment and treating participants as experts and key stakeholders in the project will ensure their inputs and suggestions are valued and incorporated into the study.

The steps taken in order to perform our study so far have broader value. These steps should be adopted by most research studies in the health care sector and are especially important in environments where it may be difficult to get buy-in regarding participation.

During this process, local health care centers commented that they required further data science education. To this end, we created a free data science workshop to teach basic skills to those working in the health care sector. As part of this workshop, we invited the participants to help us collect further data about Botswana’s data health care infrastructure as part of phase 2 of our project to try and broaden and deepen our understanding.

The data collection process commenced in June 2022 and was projected to end in December 2023. Data were collected virtually using REDCap forms, and interviews were conducted via Zoom and Microsoft Teams and then transcribed into the REDCap system. Out of the 30 facilities enrolled in the study, as of October 24, 2023, we have collected 200 records for the initial survey and captured 90 records for the semistructured interviews.

## Discussion

This research project aims to systematically map the data flow within Botswana's health care infrastructure and to delve into health care workers' perspectives on data sharing practices. Using a series of sensitization meetings, the study effectively engaged the community, making them aware of the project and eliciting feedback for the project itself. The enthusiastic engagement from the health care community underscored the relevance and necessity of the study’s objectives. The use of virtual tools (such as REDCap forms) for data collection demonstrates adaptability to modern technologies, which can enhance efficiency in remote data collection, especially in the context of global events such as the COVID-19 pandemic. As of October 24, 2023, over 200 records were collected for the initial survey and 90 records were captured for the semistructured interviews from 30 facilities around the country.

Given the response and enthusiasm to the study, it would seem to suggest both that the health care community appreciates why this project is important and that engaging the community early and fostering collaboration and transparency encourage more active participation. This approach was instrumental in building trust and a sense of ownership among participants, qualities that are often overlooked yet critical for the success of such endeavors. The preliminary results do seem to indicate that the study was successful in engaging a wide range of health care professionals involved in the day-to-day management and utilization of health care data. The insights derived will help identify bottlenecks in data flow and opportunities for enhancing interoperability among the myriad of health care information systems currently in use.

The study highlights the significance of engaging stakeholders in the health care sector, including health care professionals from various types of facilities. Sensitization meetings and workshops were found to be crucial in encouraging participation and ensuring the insights and perspectives of participants were valued. This emphasizes the importance of collaboration and inclusive decision-making processes in health care research. The finding that stakeholder engagement and participation are critical for the success of health care research aligns with previously published works. A growing body of literature in global health has emphasized the importance of involving stakeholders, including health care professionals, in all stages of research to enhance relevance, ownership, sustainability, and uphold ethical standards [30-33]. Although no studies specifically evaluating participatory research in global health informatics were found, this study would seem to suggest that these principles observed in a broader global health context appear to be applicable.

There are a number of limitations that should be taken into account. A wide range of health care professionals, but with this kind of study, a larger cross section of the health care workers would improve the generalizability of the findings. In addition, the study relied on self-reported data, which may introduce response bias. Interviews conducted and recorded by research assistants could have inconsistencies due to variations in data capturing or missing nuanced information from participants. Future research could benefit from a larger sample size, more varied health care facilities, audio recording and later transcribing all of the interviews, and direct observation at health care facilities.

As we move forward, the focus will shift toward a more in-depth analysis of the data collected, with the aim of developing comprehensive recommendations for improving data flow within Botswana's health care system. The anticipated second manuscript will detail these findings and recommendations, providing more concrete guidance for Botswana’s continued digitization of its health care system. By documenting and sharing the methodology and initial steps of our study, this research aims to provide a blueprint for similar research endeavors, emphasizing the importance of community engagement and methodological rigor. This approach will not only inform health care policy but also contribute to more effective and integrated health care practices.

## Abbreviations

EHRs: electronic health records
IRB: institutional review board
MOH: Ministry of Health
PMTCT: Prevention of Mother to Child Transmission of HIV
REDCap: Research Electronic Data Capture

## Data Availability

The data sets generated or analyzed during this study are not publicly available as Botswana law requires institutional review board approval to access all health care–related research data

## References

[1] Chrysopoulos P (2020). Hippocrates: The Greek Father of Modern Medicine. GreekReporter.com.

[2] History Learning Site. Hippocrates.

[3] Universal health coverage (UHC).

[4] Dixon BE, Pina J, Kharrazi H, Gharghabi F, Richards J (2015). What's past is prologue: a scoping review of recent public health and global health informatics literature. Online J Public Health Inform.

[5] Agarwal S, Perry HB, Long LA, Labrique AB (2015). Evidence on feasibility and effective use of mHealth strategies by frontline health workers in developing countries: systematic review. Trop Med Int Health.

[6] Fraser HSF, Biondich P, Moodley D, Choi S, Mamlin BW, Szolovits P (2005). Implementing electronic medical record systems in developing countries. Inform Prim Care.

[7] Kruse CS, Kothman K, Anerobi K, Abanaka L (2016). Adoption factors of the electronic health record: a systematic review. JMIR Med Inform.

[8] Akhlaq A, McKinstry B, Muhammad KB, Sheikh A (2016). Barriers and facilitators to health information exchange in low- and middle-income country settings: a systematic review. Health Policy Plan.

[9] Garrib A, Stoops N, McKenzie A, Dlamini L, Govender T, Rohde J, Herbst K (2008). An evaluation of the district health information system in rural South Africa. S Afr Med J.

[10] Mate KS, Bennett B, Mphatswe W, Barker P, Rollins N (2009). Challenges for routine health system data management in a large public programme to prevent mother-to-child HIV transmission in South Africa. PLoS One.

[11] Charnock V (2019). Electronic healthcare records and data quality. Health Info Libr J.

[12] Geraci A (1991). IEEE Standard Computer Dictionary: Compilation of IEEE Standard Computer Glossaries.

[13] Reisman M (2017). EHRs: the challenge of making electronic data usable and interoperable. Pharm Ther.

[14] Ndlovu K, Scott RE, Mars M (2021). Interoperability opportunities and challenges in linking mhealth applications and eRecord systems: Botswana as an exemplar. BMC Med Inform Decis Mak.

[15] Francoiose B, Innocent N, Nathan S (2019). Technical support on data quality improvement for the Botswana PMTCT programme in line with requirements of validation of EMTCT for HIV and syphilis. UNICEF.

[16] Kwape D (2019). Botswana Health Data Collaborative Roadmap Report (2020 -2024).

[17] Kwape L (2020). The eHealth Strategy of Botswana (2020-2024).

[18] Vedanthan R, Blank E, Tuikong N, Kamano J, Misoi L, Tulienge D, Hutchinson C, Ascheim DD, Kimaiyo S, Fuster V, Were MC (2015). Usability and feasibility of a tablet-based Decision-Support and Integrated Record-keeping (DESIRE) tool in the nurse management of hypertension in rural western Kenya. Int J Med Inform.

[19] Ha YP, Tesfalul MA, Littman-Quinn R, Antwi C, Green RS, Mapila TO, Bellamy SL, Ncube RT, Mugisha K, Ho-Foster AR, Luberti AA, Holmes JH, Steenhoff AP, Kovarik CL (2016). Evaluation of a mobile health approach to tuberculosis contact tracing in Botswana. J Health Commun.

[20] Theiss-Nyland K, Ejersa W, Karema C, Koné D, Koenker H, Cyaka Y, Lynch M, Webster J, Lines J (2016). Operational challenges to continuous LLIN distribution: a qualitative rapid assessment in four countries. Malar J.

[21] Campbell JI, Aturinda I, Mwesigwa E, Burns B, Santorino D, Haberer JE, Bangsberg DR, Holden RJ, Ware NC, Siedner MJ (2017). The technology acceptance model for resource-limited settings (TAM-RLS): a novel framework for mobile health interventions targeted to low-literacy end-users in resource-limited settings. AIDS Behav.

[22] Clouse K, Phillips TK, Camlin C, Noholoza S, Mogoba P, Naidoo J, Langford R, Weiss M, Seebregts CJ, Myer L (2020). CareConekta: study protocol for a randomized controlled trial of a mobile health intervention to improve engagement in postpartum HIV care in South Africa. Trials.

[23] Managerial Tools for IS4H Digital Transformation - PAHO/WHO | Pan American Health Organization.

[24] Facility hit ecosystem capability maturity model toolkit.

[25] (2021). Readiness assessment maturity model.

[26] Khoja S, Scott RE, Casebeer AL, Mohsin M, Ishaq AFM, Gilani S (2007). e-Health readiness assessment tools for healthcare institutions in developing countries. Telemed J E Health.

[27] Tools for data demand and use in the health sector? MEASURE evaluation.

[28] (2015). Data-Driven Innovation: Big Data for Growth and Well-Being.

[29] Trebble TM, Hansi N, Hydes T, Smith MA, Baker M (2010). Process mapping the patient journey: an introduction. Br Med J.

[30] Davis SLM, Digital HealthRights Project Consortium (2022). Towards digital justice: participatory action research in global digital health. BMJ Glob Health.

[31] Pratt B, Sheehan M, Barsdorf N, Hyder AA (2018). Exploring the ethics of global health research priority-setting. BMC Med Ethics.

[32] Pratt B, de Vries J (2018). Community engagement in global health research that advances health equity. Bioethics.

[33] Pratt B, Cheah PY, Marsh V (2020). Solidarity and community engagement in global health research. Am J Bioeth.

